# Identification of Differentially Expressed Genes of *Trichinella spiralis* Larvae after Exposure to Host Intestine Milieu

**DOI:** 10.1371/journal.pone.0067570

**Published:** 2013-06-28

**Authors:** Hui Jun Ren, Jing Cui, Wei Yang, Ruo Dan Liu, Zhong Quan Wang

**Affiliations:** 1 Department of Parasitology, Medical College, Zhengzhou University, Zhengzhou, China; 2 Departments of Clinical Laboratory, the First Affiliated Hospital, Zhengzhou University, Zhengzhou, China; University Claude Bernard Lyon 1, France

## Abstract

Although it has been known for many years that *T. spiralis* muscle larvae (ML) can not invade intestinal epithelial cells unless they are exposed to the intestinal milieu and activated into intestinal infective larvae (IIL), which genes in IIL are involved in the process of invasion is still unknown. In this study, suppression subtractive hybridization (SSH) was performed to identify differentially expressed genes between IIL and ML. SSH library was constructed using cDNA generated from IIL as the ‘tester’. About 110 positive clones were randomly selected from the library and sequenced, of which 33 *T. spiralis* genes were identified. Thirty encoded proteins were annotated according to Gene Ontology Annotation in terms of molecular function, biological process, and cellular localization. Out of 30 annotated proteins, 16 proteins (53.3%) had binding activity and 12 proteins (40.0%) had catalytic activity. The results of real-time PCR showed that the expression of nine genes (Ts7, Ndr family protein; Ts8, serine/threonine-protein kinase polo; Ts11, proteasome subunit beta type-7; Ts17, nudix hydrolase; Ts19, ovochymase-1; Ts22, fibronectin type III domain protein; Ts23, muscle cell intermediate filament protein OV71; Ts26, neutral and basic amino acid transport protein rBAT and Ts33, FACT complex subunit SPT16) from 33 *T. spiralis* genes in IIL were up-regulated compared with that of ML. The present study provide a group of the potential invasion-related candidate genes and will be helpful for further studies of mechanisms by which *T. spiralis* infective larvae recognize and invade the intestinal epithelial cells.

## Introduction

*Trichinella spiralis* is a parasitic nematode that infects their vertebrate host by the consumption of raw or undercooked meat from infected animals (e.g. pigs, wild animals) [1], [2]. Following their release in the stomach by digestion of meat, *T. spiralis* muscle larvae (ML) are activated by intestinal content or bile after 0.9 hour post-infection (hpi), and interacted with host intestinal epithelial cells (IECs). These activated larvae in intestine are named as “intestinal infective larvae (abbreviated as IIL)” [3]. Then, the IIL penetrate into host intestinal epithelium where they molt four times during 10–28 hpi, and mature into adults that mate and produce the next generation of larvae [4]–[6]. The life cycle of *T. spiralis* is completed when the newborn larvae invade and mature in striated muscles of the new host [7].

It is well known that invasion of host intestinal epithelium is the first step during *T. spiralis* infections. However, to date, little is known at the molecular level about how *T. spiralis* IIL recognize, invade, migrate within the intestinal epithelium and establish their intramulticellular niche. Previous studies showed that ML can not invade the IECs cultured *in vitro* unless they are exposed to the intestinal milieu and activated into the IIL [8], [9]. The activation of the larvae by intestinal content or bile is one of the most pivotal requirements for the larval invasion of IECs, but why is the procedure necessary before the invasion of IECs? What is the difference in gene expression between ML and IIL? Which genes are differentially expressed significantly in *T. spiralis* larvae during the process of their activation and interaction with IECs? Among the proteins encoded by these up-regulated genes, which are related to the invasion by the parasite? These key questions related with the mechanism of the *Trichinella* invasion of host enterocytes are unknown.

Suppression subtractive hybridization (SSH) is a highly sensitive PCR-based cDNA subtraction technique used to study gene expression changes in both physiological and pathological states [10]. This method, which combines high subtraction efficiency with an equalized representation of differentially expressed cDNAs, provides an approximately 1000-fold enrichment of low copy number genes related to defined phenotypes [11]. Moreover, it offers an opportunity to characterize changes in transcription of hundreds of genes simultaneously [10]. During the past few years, SSH has proven to be a powerful and efficient tool for analysis of differential gene expression [12]–[15], including the identification of the stage-specifically expressed genes of *T. spiralis* newborn larvae and adult worms [16].

In this study, for the first time, the differentially expressed genes in IIL compared to ML were identified using SSH, and some specifically up-regulated genes in IIL were further confirmed by real time PCR. This study will be helpful in elucidating the molecular mechanism of the invasion of IECs by *T. spiralis* larvae and better understanding the interaction between parasite and host.

## Materials and Methods

### Ethics Statement

This study was carried out in strict accordance with the National Guidelines for Experimental Animal Welfare (MOST of People’s Republic of China, 2006). All animal procedures reported herein were reviewed and approved by the Zhengzhou University Animal Care and Use Committee (Permission No. SYXK 2007-0009).

### Parasites and Experiment Animals

The isolates (ISS534) of *T. spiralis* used in this study were obtained from a domestic pig in Nanyang city of Henan Province, China. The isolate was maintained by serial passages in Kunming mice in our laboratory. Six-week-old male Kunming mice were obtained from the Experimental Animal Center of Henan Province (Zhengzhou, China). The mice were maintained under specific pathogen-free conditions with sterilized food and water.

Muscle larvae (ML) were recovered from the 50 infected male mice at 42 days post infection (dpi) by artificial digestion as described previously [17], [18]. After recovery, a part of ML were directly pooled for extracting total RNA; the other part were orally inoculated into 100 mice, with 5000 ML per mouse in a volume of 100 µl. And then, the IIL were collected from the mouse small intestines at 4 hpi. In order to get sufficient ML and IIL for SSH library construction, about one million of ML and IIL were respectively obtained. After several washes in sterile RNase-free water, all parasites were centrifuged at 600 g for 10 min and stored at −80°C until use.

### Total RNA and Poly (A)^+^ RNA Purification

Total RNA was isolated respectively from *T. spiralis* ML and IIL using TRIzol reagent (Invitrogen, USA) according to manufacturer’s instructions. Poly (A)^+^ RNA was isolated and purified from 200 µg of total RNA using an Oligotex mRNA Mini kit (Qiagen, Germany). The concentration and purity of total RNA was determined by NanoDrop 2000 spectrophotometer (Thermo Scientific Nanodrop, USA) at 260 nm and verified the ratio 260/280 nm.

### Construction of SSH Library and Sequencing

SSH was performed using a PCR-Select cDNA Subtraction kit (Clontech) according to the manufacturer’s instructions. To identify genes that were up-regulated in *T. spiralis* larvae after their exposure to intestine milieu, cDNA obtained from the IIL was used as the tester cDNA, while cDNA from ML was used as the driver cDNA. Briefly, double-stranded cDNA was produced from approximately 2 µg of poly (A)^+^ RNA. The cDNA from the tester and the driver were digested with Rsa I, and then the tester cDNA was ligated into either two different cDNA adaptors. During a first hybridization, excess driver was added to the tester cDNA samples, which were then denatured and allowed to anneal. In the second hybridization, the two primary hybridization samples were mixed without denaturation. To further select for differentially expressed sequences, denatured driver cDNA was added to these hybrid samples again. The resulting mixture was amplified by two rounds of PCR to enrich for the desired cDNAs containing both adaptors, by exponential amplification of these products. Finally, the efficiency of the cDNA subtraction was evaluated by PCR using 18S rRNA primers (GenBank Accession No. XM_003379058.1; Table 1) performed on subtracted and unsubtracted cDNAs for 18, 23, 28, and 33 cycles.

**Table 1:** Primers used in the real-time PCR assays.

Gene description (sequence origin SSH)	GenBank accession	Primer sequence	Product
	number		size (bp)
serine/threonine-protein kinase polo (Ts8)	XM_003378839.1	F 5′–TGAAGCCGAAGATCCTGCGTT–3′	94
		R 5′–GTCGCTCAACTGATAGCCAATACC–3′	
putative nudix hydrolase (Ts17)	EU263318.1	F 5′–GTTGCTGCTGAAGTCGGAAAGA–3′	161
		R 5′–AAACCCAAAGCACCAAGGACAG–3′	
Ndr family protein (Ts7)	XM_003378649.1	F 5′–TGGTCGTTGATGATGTCGG–3′	194
		R 5′–ACTTGGTGCTTTAGTCAGGGATA–3′	
muscle cell intermediate filament protein OV71 (Ts23)	XM_003372805.1	F 5′–GTCTCGCACATGAAAGCCA–3′	134
		R 5′–CCGACAACTAGCATAAAACCGT–3′	
putative fibronectin type III domain protein (Ts22)	XM_003374166.1	F 5′–TGCTTTGCCAGCTCCAC–3′	93
		R 5′–AGCCGACTAACGACCTCCT–3′	
neutral and basic amino acid transport protein rBAT (Ts26)	XM_003368418.1	F 5′–TCACCACTTCCGAGAAACAC–3′	108
		R 5′–CTGTGACGTAATAATTCCCTCC–3′	
putative ovochymase-1 (Ts19)	XM_003369378.1	F 5′–TACAAGTAGCGGCAGTAGCAG–3′	227
		R 5′–CAGGATAAGCGAAGTTAGGGA–3′	
FACT complex subunit SPT16 (Ts33)	XM_003371753.1	F 5′–CGCTTCAGTTCTCCTCCAA–3′	185
		R 5′–CAAGCATGTGCTGAGGGAT–3′	
proteasome subunit beta type-7 (Ts11)	XM_003374391.1	F 5′–CTGGAATGGCAGCGGATGT–3′	137
		R 5′–TGGATAGCGGTAAGCGAGCA–3′	
18S rRNA (Reference)	XM_003379058.1	F 5′–TGCGGCAACGATTTTGAAA–3′	296
		R 5′–CGCATTACTGGAAGCCAAGC–3′	
G3PDH (Reference)	AF452239.1	F 5′–AGATGCTCCTATGTTGGTTATGGG–3′	186
		R 5′–GTCTTTTGGGTTGCCGTTGTAG–3′	

The SSH library was constructed by ligating the subtracted cDNAs into the pED-T vector (Sinobio, China) and then transferred into *Escherichia coli* DH5α cells (Invitrogen, USA). *E. coli* cells were plated on LB agar containing ampicillin, IPTG and X-gal. The plates were incubated at 37°C overnight to obtain colonies harboring subtractive sequence fragments. Individual recombinant white colonies were picked,and grown in liquid LB broth at 37°C overnight. Then the positive colonies were amplified by M13 forward and reverse primers, and the PCR products were sequenced using an automated sequenator (Applied Biosystems, USA, and model 473A). After removal of flanking vector regions, the cDNA sequences were compared with the GenBank database using the NCBI-BLAST server (http://www.ncbi. nlm.nih.gov/BLAST).

Functional characterization of *Trichinella* sequences was based on Gene Ontology (GO) Annotation. The signatures of *Trichinella* protein sequences were queried against InterPro member databases by InterProScan searching (http://www.ebi.ac.uk/InterProScan/) [19], and the resultant proteins were functionally categorized using the WEGO [Web Gene Ontology Annotation Plot (http://wego. genomics.org.cn/cgi-bin/wego/)] [20]. Final gene annotation was based on top blast hits and GO terms.

### Quantification of Specific Transcripts by Real-time PCR

The transcription of nine genes selected from the SSH library was evaluated by real-time PCR. G3PDH was used as a reference gene to normalize gene expression [21], and there were no differences in G3PDH expression between *T. spiralis* IIL and ML (data not shown). The primers were designed using the Primer5.0 software, and details of gene specific primers were listed in Table 1. Total RNA was purified and first-strand cDNA was generated using PrimeScript RT reagent Kit gDNA Eraser (Takara, Japan) according to the manufacturer’s protocol. Both of Oligo dT primer and random 6 mers were used for reverse transcription. Generated cDNAs were diluted at 1/30 with sterile water before use. Real-time PCR was performed in total volume of 20 µl containing diluted cDNA (2 µl), 10 µl of 2× SYBR Premix Ex Taq (Takara, Japan), 0.4 µl of each primer (10 µM final concentration), 0.4 µl of 50× ROX Reference Dye II, and 6.8 µl of deionized water. PCR was run on an ABI 7500 fast real time PCR system (Applied Biosystems, USA). The cycling conditions used were 95°C for 30 s, followed by 40 cycles of 95°C for 3 s and 60°C for 30 s. A melting curve was performed to confirm amplification of specific products. A no-template control was included on each reaction plate. Relative expression levels of the target genes were normalized to G3PDH, and then calculated using comparative Ct (2^−△△Ct^) method [22]. Each sample had three replicates and each experiment was repeated three times.

### Statistical Analysis

All data are expressed as means ± standard deviation (SD) [22]. Intra- and intergroup statistical analyses were performed with one-way ANOVA (LSD test) using SPSS version 17.0 software (SPSS Inc., Chicago, IL). Differences were considered to be statistically significant at *P*<0.05.

## Results

### Evaluation of Subtraction Efficiency

The subtracted cDNAs specific for IIL were evaluated by PCR analysis using 18S rRNA gene as probe after subtractive hybridizations were performed. The amount of 18S rRNA transcript decreased significantly after subtraction. In subtracted cDNAs, 18S rRNA products were observed at 28 cycles, while the amplified products were seen at 18 cycles in the unsubtracted cDNAs. The results indicated that existence of the 18S rRNA gene was reduced by up to 2^10^-fold after subtraction, suggesting that the subtraction procedure was successful, and suppression subtractive hybridization between IIL and ML has effectively excluded non-stage specific expression genes.

### Characterization of the Subtracted cDNA Library

The subtracted cDNA library which was generated in this study contained the up-regulated genes in *Trichinella* larvae after their exposure to mouse intestine milieu. Following cloning and transformation, 122 bacterial clones were isolated and amplified by PCR. The results showed that 5.7% (7/122) of these clones failed to give amplification products with more than 100bp, while 4.1% (5/122) of the clones resulted in multiple amplifications. A total of 110 recombinant clones were sequenced, and then 61 qualified sequences with insertions longer than 100 bp were conducted for the sequence homology searching against GenBank using BLAST. These search results revealed that out of the 61 positive clones, 33 represented unique *T. spiralis* genes, while 28 were duplicates. Of the 33 unique genes, 29 showed high homologous to known proteins, while the remaining 4 were hypothetical proteins (Table 2).

**Table 2:** The identified up-regulated genes of *T. spiralis* after exposure to mouse intestine milieu.

GenBank	Seq.	Homologous genes	Seq. length	BLASTN	Id	N	Putative molecular function
Accession No.	name			E value	(%)		
JZ077049	Ts3	*T. spiralis* serine proteinase	536	0.0	100	3	peptidase activity
JZ077065	Ts19	*T. spiralis* putative ovochymase-1	290	3e-149	100	3	peptidase activity
JZ077057	Ts11	*T. spiralis* proteasome subunit beta type-7	170	8e-83	100	2	peptidase activity
JZ077054	Ts8	*T. spiralis* serine/threonine-protein kinase polo	239	5e-121	100	4	transferase activity; protein binding; ATP binding
JZ077050	Ts4	*T. spiralis* adenosylhomocysteinase	965	0.0	99	1	hydrolase activity, acting on ether bonds
JZ077063	Ts17	*T. spiralis* putative nudix hydrolase	191	2e-94	100	5	hydrolase activity
JZ077076	Ts30	*T. spiralis* DNA topoisomerase 3-beta-1 protein	274	5e-137	99	2	isomerase activity; DNA binding
JZ077071	Ts25	*T. spiralis* acetyl-CoA carboxylase	505	0.0	100	2	acetyl\-CoA carboxylase activity; metal ion binding; ATP binding
JZ077061	Ts15	*T. spiralis* ubiquitin-conjugating enzyme E2 1	391	0.0	99	1	ligase activity
JZ077068	Ts22	*T. spiralis* putative fibronectin type III domain protein	436	0.0	100	2	transmembrane transporter activity; protein binding
JZ077060	Ts14	*T. spiralis* vesicle-associated membrane protein 3	492	0.0	99	1	vesicle\-mediated transport
JZ077067	Ts21	*T. spiralis* putative transposase	269	1e-132	99	2	nucleic acid binding
JZ077051	Ts5	*T. spiralis* putative integrase core domain protein	360	2e-171	97	2	nucleic acid binding
JZ077072	Ts26	*T. spiralis* neutral and basic amino acid transport protein rBAT	210	3e-103	99	2	nucleic acid binding; cation binding
JZ077073	Ts27	*T. spiralis* protein POP2	275	5e-137	99	1	nucleic acid binding
JZ077078	Ts32	*T. spiralis* MYOD-like DNA-binding protein	329	5e-169	99	2	DNA binding
JZ077077	Ts31	*T. spiralis* cuticle collagen 1	294	2e-151	100	2	DNA binding
JZ077058	Ts12	*T. spiralis* CCR4-NOT transcription complex subunit 3	452	0.0	100	2	transcription factor activity
JZ077069	Ts23	*T. spiralis* muscle cell intermediate filament protein OV71	250	2e-125	99	2	signal transducer activity; transcription factor activity; protein binding
JZ077048	Ts2	*T. spiralis* putative proteasomal ATPase-associated factor 1	275	6e-141	100	1	protein binding
JZ077059	Ts13	*T. spiralis* glutathione S-transferase 2	240	7e-120	99	3	protein binding
JZ077055	Ts9	*T. spiralis* putative leucine Rich repeat-containing domain protein	211	8e-104	99	1	protein binding
JZ077056	Ts10	*T. spiralis* ankyrin repeat domain-containing protein 10	216	3e-108	100	1	protein binding
JZ077047	Ts1	*T. spiralis* tether containing UBX domain for GLUT4	183	3e-88	99	2	protein binding
JZ077064	Ts18	*T. spiralis* heat shock protein 90	347	1e-173	99	3	unfolded protein binding; ATP binding
JZ077070	Ts24	*T. spiralis* putative LIM domain protein	243	3e-123	100	1	zinc ion binding
JZ077079	Ts33	*T. spiralis* FACT complex subunit SPT16	455	0.0	100	1	protein binding
JZ077052	Ts6	*T. spiralis* hypothetical protein	185	2e-89	99	1	Unknown
JZ077053	Ts7	*T. spiralis* Ndr family protein	619	0.0	99	2	Unknown
JZ077062	Ts16	*T. spiralis* glyco protein hormone beta-5	682	0.0	100	1	Unknown
JZ077066	Ts20	*T. spiralis* hypothetical protein	254	3e-124	99	1	Unknown
JZ077074	Ts28	*T. spiralis* conserved hypothetical protein	200	2e-99	100	1	Unknown
JZ077075	Ts29	*T. spiralis* conserved hypothetical protein	330	9e-170	99	1	Unknown

Seq. name: code of EST sequence; Seq. length: EST length (base pair); Id (%): EST similarity; N: EST number.

**Figure 1:**
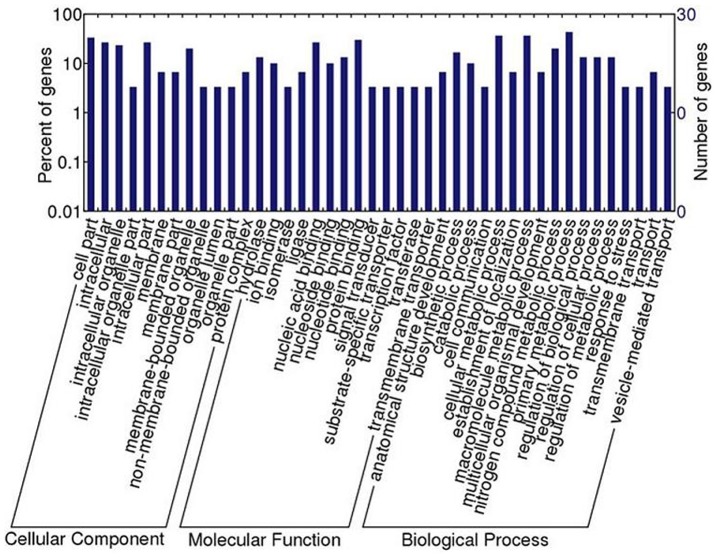
Gene ontology categories for the up-regulated proteins of *T.* *spiralis* larvae after their exposure to mouse intestine milieu. The identified proteins were classified into cellular component, molecular function, and biological process by WEGO according to their GO signatures. The number of genes denotes that of proteins with GO annotations.

To further understand the protein functions, the identified 33 protein sequences were putatively annotated using GO terms obtained from the first 20 BLAST hits or/and from protein domains obtained from the InterPro database, and their putative molecular functions were showed in Table 2. In addition, the 33 proteins were functionally categorized into cellular component, molecular function, and biological process according to GO hierarchy using WEGO. Since GO signatures of 30 out of the 33 proteins were available, Figure 1 showed the classification results of the 30 proteins identified in this study. In the cellular component category, a vast majority of the proteins were involved in “cell” (13, 43.3%) and “organelle” (19, 63.3%). Within the molecular function category, “binding” (16, 53.3%) and “catalytic activity” (12, 40.0%) were predominant. With regard to biological process, “metabolic process” (13, 43.3%), “biological regulation” (12, 40.0%), and “cellular process” (12, 40.0%) were the most frequent categories.

### Real-time PCR

**Figure 2:**
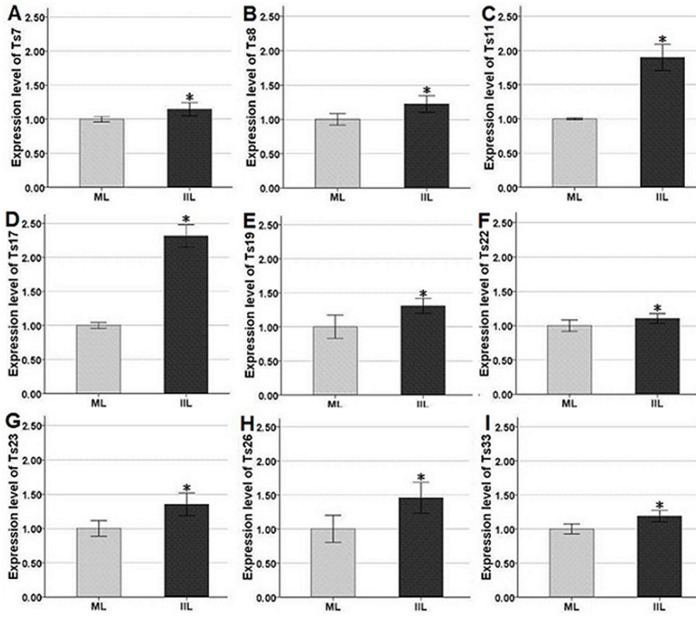
Relative expression by real-time PCR of the selected nine genes in *T.* *spiralis* larvae. Total RNAs from muscle larvae (ML) and intestinal infective larvae (IIL) were subjected to real-time PCR as described in “Material and Methods.” Expression levels were normalized to the G3PDH gene and presented as relative expression to controls (mean ± SD, n = 9). Controls are arbitrarily assigned to a value of 1. *Significant difference of gene expression compared to controls.

In order to confirm that genes identified by SSH are differentially expressed in IIL compared to ML, real-time PCR was conducted in parallel to verify the validity of the SSH data. Nine genes (Ts7, Ts8, Ts11, Ts17, Ts19, Ts22, Ts23, Ts26, and Ts33) of 33 *T. spiralis* genes identified from SSH library were randomly selected, and the expression of these genes was determined by real-time PCR using specific sets of primers. The transcription levels of mRNA were obviously increased for all the tested nine genes in IIL, compared with ML (controls) (Figure 2). The expression patterns obtained by real-time PCR reflected the results obtained by SSH, demonstrating a low false positive rate associated with SSH in this experiment.

## Discussion

Subtractive hybridization and differential display have been extensively applied to the isolation and identification of the disease-related genes, embryonic development stage-specifically expressed genes and genes that determine cell differentiation or organogenesis. Traditional subtractive hybridization methods required several rounds of hybridization and are not well suited for the identification of the unobvious differentially-expressed genes between two tissues [23], [24]. Based on the application of selective amplification of differentially expressed sequences, SSH leads to enrichment specific-expression library, besides overcoming technical limitation of traditional subtraction methods [10]. Our results showed that non-specific expression gene 18S rRNA had been reduced about 2^10^ fold after subtraction between ML and IIL. It is suggested that non-specific expressed gene cDNAs had been removed and stage-specific expressed gene cDNAs had been enriched efficiently in SSH library constructed in this study. Therefore, such a SSH library was successful. As shown in our results, 33 up-regulated genes in *T. spiralis* larvae after their exposure to mouse intestine milieu could be obtained from the library. Thus, construction of the subtractive libraries will be useful for understanding the mechanisms in the larval invasion of IECs and the establishment of their intramulticellular niche in the intestine.

*T. spiralis* is a parasite that has a relatively short inter-stage phase inside mammalian hosts with the exception of the ML, which poses challenges for targeting genes that are specifically up-regulated during certain developmental stages. In recent years, the SSH technique has been used to identify genes encoding heat shock proteins of *T. spiralis* infective larvae [25]. A clone encoding protein *Ts*-CCG-1 with a nematode-specific cysteine-glycine domain was obtained from a subtracted cDNA library of *T. spiralis* ML, and showed 90% sequence identity to a homologous gene of *T. pseudospiralis* [26]. In addition, four stage-specifically expressed genes of 3-day-old adult worms of *T. spiralis* were identified by the SSH technique [16].

In the present study, the IIL were collected at 4 hpi. The exposure of larvae to host small intestine for 4 h could make them to be activated sufficiently and interacted with intestinal epithelium [8]. Moreover, some differentially expressed genes in IIL at this time might be related to invasion of intestinal epithelium by *T. spiralis*. The results of sequence analysis showed that a total of 33 proteins were identified by SSH. Thirty of these proteins were involved in various biological processes, such as protein metabolic process, signal transduction, and biological regulation. However, out of these thirty genes, three (Ts7, Ts16, and Ts28) are still not annotated in GO for the molecular function. As shown in Table 2, six proteins have no putative molecular functions, and additional experiments are needed to elucidate their functions. These results showed that a wide variety of proteins were up-regulated in *T. spiralis* larvae, including some enzymes that are released into the environment by the parasite and might be related to the larval invasion of host enterocytes. Although further studies are needed to characterize functionally these proteins, our data provide a global view of the up-regulated genes in *T. spiralis* larvae after they are exposed to host small intestine milieu.

With regard to the intestinal stage of infection, it has been suggested that proteases participate in intestinal invasion by *T. spiralis* [27]. Previous studies have showed that some proteases (such as serine, cysteine and metalloproteases) in the ES products of *T. spiralis* ML possess collagenolytic and elastolytic activities and may play an important role in the invasion and developmental process of *Trichinella* larvae [28], [29]. In parasites, serine proteases are known to be involved in host tissue and cell invasion and are likely to be important in the molting of nematodes [30]. Several secreted serine proteases are members of the serine protease family and have been identified in the ES proteins of *T. spiralis* larvae using proteomic analyses [31]–[34]. In this study, three proteins with proteolytic activity (serine proteinase, ovochymase-1, and proteasome subunit beta type-7) were identified from the SSH library. They were found to be significantly up-regulated after *T. spiralis* larvae were exposed to mouse intestine milieu. These results suggested that the serine proteases might be related with the invasion of IECs by the infective larvae and might mediate or facilitate the entry into cells. Although the other two proteins (ovochymase-1, and proteasome subunit beta type-7) are related to peptidase activity, their exact biological functions have not been clarified. Therefore, further experiments in vitro and in vivo are needed to determine whether these proteases are related with the larval invasion of IECs.

The classification results of the 30 *Trichinella* genes showed that eight proteins have protein-binding activity. They were tether containing UBX domain for GLUT4 (Ts1), proteasomal ATPase-associated factor 1 (Ts2), leucine Rich repeat-containing domain protein (Ts9), ankyrin repeat domain-containing protein (Ts10), glutathione S-transferase 2 (Ts13), heat shock protein 90 (Ts18), muscle cell intermediate filament protein OV71 (Ts23), and FACT complex subunit SPT16 (Ts33), respectively. These proteins might be expressed on the exterior of the parasite and be available for interaction with the host cells, and involved in the process of invasion, they might bind to important structural components of the enterocyte membrane or reorganize the enterocyte skeleton during invasion. Certainly, these proteins might also be involved in developmental events, such as tissue formation, biosynthetic process, and response to stress. Hence, this hypothesis needs to be verified in further experiments.

Protein kinase (serine/threonine-protein kinase) was also identified by SSH from *T. spiralis* IIL. Protein kinases are a superfamily of enzymes, and they are involved in cell signaling pathways and signal transduction through phosphorylation/dephosphorylation of target proteins. It has been demonstrated that viruses including herpesviruses are able to modulate host cell signaling pathways. Hale and Randall [35] reported that binding of NS1 protein of influenza A virus induced an activation of phosphoinositide 3-kinase in virus-infected cells. It is still unclear whether the serine/threonine- protein kinase plays a role in *T. spiralis* invasion.

Two kinds of proteins related to stress resistance were highly expressed in *T. spiralis* IIL. One is glutathione S-transferase (GST, Ts13), and the other is heat shock protein (HSP, Ts18). GSTs are a family of multifunctional enzymes involved in detoxification of xenobiotics. The different GST enzymes have classically been viewed as part of cell defense against numerous harmful chemicals produced endogenously and in the environment [36]. So, it was suggested that GST might be related with the niche establishment by *T. spiralis* in small intestinal epithelium. HSPs are produced as an adaptive response of the parasite to the hostile environment encountered in the host intestine [37], [38]. They are involved in binding antigens and presenting them to the immune system [39]. Recent studies suggested that HSPs are implicated in disease development, proliferation and regulation of cancer cell, cell death via apoptosis, and several other key cellular functions [40]. In this study, Hsp90 was significantly up-regulated in *T. spiralis* larvae exposed to the small intestine milieu, though its role in this process is unknown.

Additionally, our other study indicated that out of the 9 up-regulated *T. spiralis* genes conformed by real-time PCR, four gene-encoded proteins (nudix hydrolase, ovochymase-1, conserved hypothetical protein, and FACT complex subunit SPT16) were found specifically to bind to normal mouse IECs [41]. Future experiments will be necessary to determine whether these up-regulated *T. spiralis* genes play important roles in the recognition and invasion of host IECs.

Nudix hydrolases are found in all classes of organism and hydrolyse a wide range of organic pyrophosphates, including nucleoside di- and triphosphates, dinucleoside and diphosphoinositol polyphosphates, nucleotide sugars and RNA caps, with varying degrees of substrate specificity [42]. Some superfamily members, such as MutT, are known to have the ability to degrade potentially mutagenic, oxidized nucleotides, while others control the levels of metabolic intermediates and signaling compounds [42]. A previous study showed that *Escherichia coli* NudH is contributed to invasion of human brain microvascular endothelial cell by *E. coli* [43]. Human NUDT14 participates in the regulation of Raf signal transduction pathway and plays important roles in the reorganization of cytoskeleton and the changes of cell morphology [44]. In our previous studies, the antibodies against *T. spiralis* nudix hydrolase prevented the *in vitro* partial larval invasion of IECs and development [45], and the mice immunized with the recombinant phage (T7-nudix hydrolase polypeptides) showed a 62.8% reduction in adult worms following challenge with *T. spiralis* muscle larvae [46]. It is suggested that nudix hydrolase might bind to important structural components (Raf) of the enterocyte membrane and reorganize the cytoskeleton so as to mediate invasion by *T. spiralis*. This may be one of the possible reasons why the mice vaccinated with T7-Tsp10 showed a significant reduction of adult worms in intestines.

In conclusion, SSH is a useful technique for identification of stage-specifically expressed genes of *T. spiralis*. Our results provide an initial characterization of a set of genes with diverse functions that are up-regulated when *T. spiralis* ML are exposed to host intestine milieu and activated into IIL. Although the protein expression of these genes and their biological significance requires further study, the results provide a group of candidate genes and will pave the way for further study of mechanisms involving parasite invasion.

## References

[1] Pozio E, Hoberg E, La Rosa G, Zarlenga DS (2009) Molecular taxonomy, phylogeny and biogeography of nematodes belonging to the *Trichinella* genus. Infect Genet Evol 9: 606–616. 19460327 10.1016/j.meegid.2009.03.003

[2] Murrell KD, Pozio E (2011) Worldwide occurrence and impact of human trichinellosis, 1986–2009. Emerg Infect Dis 17: 2194–2202. 22172230 10.3201/eid1712.110896 PMC3311199

[3] Despommier DD (1983) *Trichinella* and Trichinosis. Plenum Press, New York. 75 p.

[4] Kozek WJ (1971) The molting pattern in *Trichinella spiralis*. I. A light microscope study. J Parasitol 57: 1015–1028. 5133876

[5] Kang SA, Cho MK, Park MK, Kim DH, Hong YC, et al (2012) Alteration of helper T-cell related cytokine production in splenocytes during *Trichinella spiralis* infection. Vet Parasitol 186: 319–327. 22222009 10.1016/j.vetpar.2011.12.002

[6] Ali Khan Z (1966) The postembryonic development of *Trichinella spiralis* with special reference to ecdysis. J Parasitol 52: 248–259.

[7] Martinez J, Rodriguez-Caabeiro F (2005) Relationship between heat shock protein levels and infectivity in *Trichinella spiralis* larvae exposed to different stressors. Parasitol Res 97: 213–218. 15997408 10.1007/s00436-005-1420-9

[8] ManWarren T, Gagliardo L, Geyer J, McVay C, Pearce-Kelling S, et al (1997) Invasion of intestinal epithelia in vitro by the parasitic nematode *Trichinella spiralis* . Infect Immun 65: 4806–4812. 9353069 10.1128/iai.65.11.4806-4812.1997 PMC175690

[9] Wang L, Cui J, Wang SW, Wang ZQ (2010) Observation of the in vitro invasion of intestinal epithelial cells by *Trichinella spiralis* larvae and their development in those cells. J Pathog Biol: 901–903.

[10] Diatchenko L, Lau YF, Campbell AP, Chenchik A, Moqadam F, et al (1996) Suppression subtractive hybridization: a method for generating differentially regulated or tissue-specific cDNA probes and libraries. Proc Natl Acad Sci U S A 93: 6025–6030. 8650213 10.1073/pnas.93.12.6025 PMC39182

[11] Qin M, Zeng Z, Zheng J, Shah PK, Schwartz SM, et al (2003) Suppression subtractive hybridization identifies distinctive expression markers for coronary and internal mammary arteries. Arterioscler Thromb Vasc Biol 23: 425–433. 12615697 10.1161/01.ATV.0000059303.94760.5C PMC3579564

[12] Reyes A, Salazar M, Granja C (2007) Temperature modifies gene expression in subcuticular epithelial cells of white spot syndrome virus-infected *Litopenaeus vannamei* . Dev Comp Immunol 31: 23–29. 16814383 10.1016/j.dci.2006.05.003

[13] Venkatachalam P, Jain A, Sahi S, Raghothama K (2009) Molecular cloning and characterization of phosphate (Pi) responsive genes in Gulf ryegrass (Lolium multiflorum L.): a Pi hyperaccumulator. Plant Mol Biol 69: 1–21. 18821059 10.1007/s11103-008-9401-x

[14] Renault T, Faury N, Barbosa-Solomieu V, Moreau K (2011) Suppression substractive hybridisation (SSH) and real time PCR reveal differential gene expression in the Pacific cupped oyster, *Crassostrea gigas*, challenged with Ostreid herpesvirus 1. Dev Comp Immunol 35: 725–735. 21371503 10.1016/j.dci.2011.02.004

[15] Xu T, Huang W, Zhang X, Ye B, Zhou H, et al (2012) Identification and characterization of genes related to the development of breast muscles in Pekin duck. Mol Biol Rep 39: 7647–7655. 22451153 10.1007/s11033-012-1599-7

[16] Liu MY, Wang XL, Fu BQ, Li CY, Wu XP, et al (2007) Identification of stage-specifically expressed genes of *Trichinella spiralis* by suppression subtractive hybridization. Parasitology 134: 1443–1455. 17475093 10.1017/S0031182007002855

[17] Gamble HR, Bessonov AS, Cuperlovic K, Gajadhar AA, van Knapen F, et al (2000) International Commission on Trichinellosis: recommendations on methods for the control of *Trichinella* in domestic and wild animals intended for human consumption. Vet Parasitol 93: 393–408. 11099850 10.1016/s0304-4017(00)00354-x

[18] Li F, Cui J, Wang ZQ, Jiang P (2010) Sensitivity and optimization of artificial digestion in the inspection of meat for *Trichinella spiralis* . Foodborne Pathog Dis 7: 879–885. 20524897 10.1089/fpd.2009.0445

[19] Mulder N, Apweiler R (2007) InterPro and InterProScan: tools for protein sequence classification and comparison. Methods Mol Biol 396: 59–70. 18025686 10.1007/978-1-59745-515-2_5

[20] Ye J, Fang L, Zheng H, Zhang Y, Chen J, et al (2006) WEGO: a web tool for plotting GO annotations. Nucleic Acids Res 34: W293–297. 16845012 10.1093/nar/gkl031 PMC1538768

[21] Chen AQ, Wang ZG, Xu ZR, Yu SD, Yang ZG (2009) Analysis of gene expression in granulosa cells of ovine antral growing follicles using suppressive subtractive hybridization. Anim Reprod Sci 115: 39–48. 19211204 10.1016/j.anireprosci.2008.10.022

[22] Schmittgen TD, Livak KJ (2008) Analyzing real-time PCR data by the comparative C (T) method. Nat Protoc 3: 1101–1108. 18546601 10.1038/nprot.2008.73

[23] Hara E, Kato T, Nakada S, Sekiya S, Oda K (1991) Subtractive cDNA cloning using oligo(dT)30-latex and PCR: isolation of cDNA clones specific to undifferentiated human embryonal carcinoma cells. Nucleic Acids Res 19: 7097–7104. 1766870 10.1093/nar/19.25.7097 PMC332523

[24] Hedrick SM, Cohen DI, Nielsen EA, Davis MM (1984) Isolation of cDNA clones encoding T cell-specific membrane-associated proteins. Nature 308: 149–153. 6199676 10.1038/308149a0

[25] Mak Ch, Su KW, Ko RC (2001) Identification of some heat-induced genes of *Trichinella spiralis* . Parasitology 123: 293–300. 11578093 10.1017/s0031182001008320

[26] Gare D, Boyd J, Connolly B (2004) Developmental regulation and secretion of nematode-specific cysteine-glycine domain proteins in *Trichinella spiralis* . Mol Biochem Parasitol 134: 257–266. 15003845 10.1016/j.molbiopara.2004.01.001

[27] de Armas-Serra C, Gimenez-Pardo C, Bernadina WE, Rodriguez-Caabeiro F (1995) Antibody response to a protease secreted by *Trichinella spiralis* muscle larvae. Parasitol Res 81: 540–542. 7567916 10.1007/BF00931800

[28] Bolás-Fernandez F, Corral-Bezara LD (2006) TSL-1 antigens of *Trichinella*: an overview of their potential role in parasite invasion, survival and serodiagnosis of trichinellosis. Res Vet Sci 81: 297–303. 16516940 10.1016/j.rvsc.2006.01.002

[29] Inaba T, Sato H, Kamiya H (2003) Monoclonal IgA antibody-mediated expulsion of *Trichinella* from the intestine of mice. Parasitology 126: 591–598. 12866798 10.1017/s003118200300310x

[30] Dzik JM (2006) Molecules released by helminth parasites involved in host colonization. Acta Biochim Pol 53: 33–64. 16410836

[31] Romaris F, North SJ, Gagliardo LF, Butcher BA, Ghosh K, et al (2002) A putative serine protease among the excretory-secretory glycoproteins of L1 *Trichinella spiralis* Mol Biochem Parasitol. 122: 149–60. 10.1016/s0166-6851(02)00094-4 12106869

[32] Robinson MW, Connolly B (2005) Proteomic analysis of the excretory-secretory proteins of the *Trichinella spiralis* L1 larva, a nematode parasite of skeletal muscle. Proteomics 5: 4525–4532. 16220533 10.1002/pmic.200402057

[33] Wang ZQ, Wang L, Cui J (2012) Proteomic analysis of *Trichinella spiralis* proteins in intestinal epithelial cells after culture with their larvae by shotgun LC-MS/MS approach. J Proteomics 75: 2375–2383. 22348823 10.1016/j.jprot.2012.02.005

[34] Wang L,Wang ZQ, Cui J (2013) Proteomic analysis of the changed proteins of *Trichinella spiralis* infective larvae after co-culture in vitro with intestinal epithelial cells. Vet Parasitol, http://dx.doi.org/10.1016/j.vetpar.2013.01.045. 10.1016/j.vetpar.2013.01.045 23433641

[35] Hale BG, Randall RE (2007) PI3K signalling during influenza A virus infections. Biochem Soc Trans 35: 186–187. 17371234 10.1042/BST0350186

[36] Strange RC, Spiteri MA, Ramachandran S, Fryer AA (2001) Glutathione-S-transferase family of enzymes. Mutat Res 482: 21–26. 11535245 10.1016/s0027-5107(01)00206-8

[37] Vayssier M, Le Guerhier F, Fabien JF, Philippe H, Vallet C, et al (1999) Cloning and analysis of a *Trichinella* britovi gene encoding a cytoplasmic heat shock protein of 72 kDa. Parasitology 119: 81–93. 10446707 10.1017/s0031182099004461

[38] Wang SA, Chuang JY, Yeh SH, Wang YT, Liu YW, et al (2009) Heat shock protein 90 is important for Sp1 stability during mitosis. J Mol Biol 387: 1106–1119. 19245816 10.1016/j.jmb.2009.02.040

[39] Nishikawa M, Takemoto S, Takakura Y (2008) Heat shock protein derivatives for delivery of antigens to antigen presenting cells. Int J Pharm 354: 23–27. 17980980 10.1016/j.ijpharm.2007.09.030

[40] Manjunatha HB, Rajesh RK, Aparna HS (2010) Silkworm thermal biology: a review of heat shock response, heat shock proteins and heat acclimation in the domesticated silkworm, *Bombyx mori* . J Insect Sci 10: 204. 21265618 10.1673/031.010.20401 PMC3029153

[41] Ren HJ, Liu RD, Wang ZQ, Cui J (2013) Construction of a *Trichinella spiralis* phage display library and use for identifying the host enterocyte-parasite interactions. Parasitol Res. DOI: 10.1007/s00436-013-3339-x 10.1007/s00436-013-3339-x 23420409

[42] McLennan AG (2006) The Nudix hydrolase superfamily. Cell Mol Life 63: 123–43. 10.1007/s00018-005-5386-7 PMC11136074 16378245

[43] Badger JL, Wass CA, Kim KS (2000) Identification of *Escherichia coli* K1 genes contributing to human brain microvascular endothelial cell invasion by differential fluorescence induction. Mol Microbiol 36(1): 174–82. 10760174 10.1046/j.1365-2958.2000.01840.x

[44] Yuryev A, Wennogle LP (2003) Novel raf kinase protein-protein interactions found by an exhaustive yeast two-hybrid analysis. Genomics 81(2): 112–25. 12620389 10.1016/s0888-7543(02)00008-3

[45] Ren HJ, Jing FJ, Liu RD, Wang B, Wang ZQ, et al..(2013) Effect of Tsp10 polypeptide immune sera on *in vitro* invasion of intestinal epithelial cells by *Trichinella spiralis* and larval development. Chin J Zoonoses, 29, in press.

[46] Cui J, Ren HJ, Liu RD, Wang L, Zhang ZF, et al (2013) Phage-displayed specific polypeptide antigens induce significant protective immunity against *Trichinella spiralis* infection in BALB/c mice. Vaccine 31: 1171–1178. 23306358 10.1016/j.vaccine.2012.12.070

